# The Impact of COVID-19 on Mental Health: The Role of Locus on Control and Internet Use

**DOI:** 10.3390/ijerph17196985

**Published:** 2020-09-24

**Authors:** Rannveig Sigurvinsdottir, Ingibjorg E. Thorisdottir, Haukur Freyr Gylfason

**Affiliations:** 1 Department of Psychology, Reykjavik University, 102 Reykjavik, Iceland; ingibjorg.eva@gmail.com; 2 Icelandic Centre for Social Research and Analysis, 102 Reykjavik, Iceland; 3 Department of Business, Reykjavik University, 102 Reykjavik, Iceland; haukurgy@ru.is

**Keywords:** COVID-19, mental health, internet use, locus of control

## Abstract

The true extent of the mental health implications of the COVID-19 pandemic are unclear, but early evidence suggests poorer mental health among those exposed to the pandemic. The Internet may have differential effects, by both connecting people with resources, or reinforce the constant checking of negative information. Moreover, locus of control becomes important in an uncontrollable pandemic. The current study aimed to examine whether exposure to COVID-19 would relate to greater symptoms of depression, anxiety and stress, and to examine the role of internet use and locus of control. Adults in the United States and five European countries (N = 1723) answered an online survey through the website Mturk. Results show elevated psychological symptoms among those who have become infected with COVID-19 or perceive themselves to be at high risk if infected. Experience using the Internet relates to fewer symptoms, but information seeking is associated with more symptoms. Internet social capital relates to fewer symptoms of depression. Having an external locus of control relates to greater symptoms. These findings suggest that public health officials need to focus on the mental health effects of the pandemic, and that internet use and locus of control could be targets to improve mental health in the population.

## 1. Introduction

The COVID-19 pandemic is currently having unprecedented effects on the physical health of individuals worldwide, with millions of confirmed cases and hundreds of thousands of deaths at the writing of this paper [1]. However, its mental health implications remain unclear. Early evidence from China suggests that, during the pandemic, the general public has reported elevated symptoms of anxiety, depression [2,3], stress [3] and increased psychological distress [4]. For example, a Hong Kong study showed that 19% of participants met the criteria for a depressive disorder, and 14% for an anxiety disorder [5]. Longitudinal evidence shows that since the pandemic began, individuals have reported greater emotional distress [6], more negative effects, as well as higher symptoms of depression and anxiety [7]. When asked retrospectively, 25.4% of the Chinese general public reported feeling that their mental health had deteriorated since the pandemic began [5]. Elevated mental health problems are also present among adults in the United Kingdom [8], Spanish university students and staff [9] and adults in the United States, where 43.3% reported high levels of depression and 45.4% reported high levels of anxiety [10]. Similarly, in Denmark, the general population’s psychological well-being was lower during the COVID-19 pandemic, compared to data collected in 2016 [11].

Greater exposure to COVID-19, such as being infected, is related to higher symptoms of stress, depression and anxiety [12]. For example, following a COVID-19 diagnosis, individuals report greater distress [13] and more mental health problems [14]. A systematic review showed that upon COVID-19 hospital admission, symptoms of anxiety and depression were common, and they persisted in the post-illness stage [15], consistent with evidence from previous epidemics where individuals suffer from social and mental health problems after they have recovered from the virus, such as post-SARS [16] or post-Ebola [17,18].

The infection of friends and family may also result in decreased well-being, as evidenced by higher anxiety levels among Chinese college students who had friends or relatives infected with COVID-19 [19]. Some may also perceive themselves at high risk if infected, such as the elderly or those with compromised immune systems. The risk is indeed higher, as preliminary data from South Korea, Spain, Italy and China show a death rate of 13–20% for individuals over 80, but less than one percent for those under the age of 50 [20]. Worries associated with getting infected can themselves be detrimental to mental health, as they relate to more anxiety and depression [5], even up to clinical levels of those disorders [10].

Given the potentially severe implications of COVID-19 on mental health, identifying moderating factors becomes essential. In times of social distancing, the Internet has started to play an ever larger role in people’s lives, but the effects of the Internet differ by activities. Internet efficacy is how confident people feel in using the Internet to find information, but general self-efficacy relates to more positive mental health [21], suggesting that greater internet efficacy could be helpful in promoting mental health. Unsurprisingly, internet efficacy is also related to greater experience using the Internet [22]. However, internet efficacy is also related to more information seeking [22], which is associated with greater anxiety [23]. For example, individuals who look at medical websites report greater anxiety sensitivity than a control group, which is a predisposing factor for anxiety psychopathology [24]. An analysis revealed that blogs related to the pandemic H1N1 were also characterized by greater anxiety than control blogs, and such posts preceded looking up information about the disease on Wikipedia [25]. In the context of COVID-19, among parents in Bahrain, the frequency of seeking out pandemic information was related to greater anxiety symptoms [26]. More media exposure can also be harmful, as seen during the Ebola outbreak, where the United States had few cases, but individuals with more media exposure reported more distress [27]. For COVID-19, very frequent media exposure was related to greater mental health problems among Chinese adults [14]. Another study found that greater symptoms of anxiety, depression, and stress were only related to the use of new media (internet and social media), but not for traditional media consumption [28]. Social media use (including seeking information on social networking services) is also related to greater depression [29], greater anxiety [30] and greater mental distress [31]. These potentially detrimental effects of information seeking could perhaps be due to having constant access to information about the pandemic through the Internet, which could have harmful effects [4,32], as well as traditional media not being updated throughout the day with multiple stories on how the disease manifests. The potential harm of information seeking can also be seen among young adults, who are more likely to use the Internet to access information and are more susceptible to psychological symptoms during COVID-19 [2,4,8,13,32].

On the other hand, the Internet could also promote positive mental health by connecting us with other people and resources during social distancing and quarantine. Social capital is the actual or potential resources available to an individual, and includes belonging, trust, and participation. It relates to a broad range of improved health outcomes, such as lower depression, better self-rated health, and more health behaviors [33]. In the current pandemic, Chinese adults who self-isolated for 14 days reported that social capital was protective against anxiety and stress [34]. Traditionally, social capital refers to in-person connections, such as meeting friends and family and participating in community activities. However, COVID-19 has limited such activities, shifting the focus instead to online social capital and its effects. For example, online social capital was related to greater well-being among college students in the United States [35]. A Korean panel study also found a harmful feedback loop, where those with low internet social capital reported greater depression, which then predicted a greater use of social networking sites. However, those with higher online social capital experienced a larger network of people, which predicted better life satisfaction [36]. Perhaps no single study encapsulates the complex effects of the Internet on mental health better than one carried out with a nationally representative sample in China, which found that using a mobile phone to communicate with others promoted greater subjective well-being, but using the mobile phone to look for information was related to more negative effects [37]. The mental health implications of online social capital need to be better understood in the context of COVID-19, but these studies suggest that it could be protective against mental health problems.

The mental health impact of COVID-19 may also depend on the locus of control, which is to what extent people believe that they control their own lives (internal locus) or whether other forces are in control (external locus, such as the influence of powerful others or by chance). Having an internal locus of control is beneficial to mental health, such as symptoms of depression [38,39]. In the context of a global pandemic, locus of control may be particularly important to determine health outcomes. Individuals with a strong internal locus of control may be more likely to take precautionary measures and believe that their actions matter. However, those who feel less in control could internalize more the threat of the pandemic, which may lead to negative feelings. To our knowledge, no studies exist on locus of control in the context of COVID-19, but some evidence suggests that a sense of control relates to greater happiness and well-being [6]. Locus of control may also connect to internet use, information seeking and online social capital, which may be higher among those with an internal locus. Studies have shown positive correlations between internal locus of control and health information seeking behavior [40]. Furthermore, studies looking especially at internal health locus of control have found that those with high internal locus are more likely to search for health-related information [41]. However, searching for online health information can have both positive and negative effects, as online health information seeking has been found to be related to increased emotional distress, as well as encouraging health promoting activities. Internet efficacy in the form of ehealth literacy can be a protective factor against emotional distress associated with information seeking online, with stronger effects for those with low internal health locus of control [42]. A further understanding of how locus of control relates to mental health and the role of internet efficacy and internet experience in that relationship is needed. It could be that seeking information and using technology help to promote a sense of control, but this is yet to be tested.

### Current Study

Previous literature suggests that COVID-19 has adverse effects on mental health and that this depends on exposure to the pandemic. The few studies already conducted on the topic have mostly been descriptive [2,3,4,5,6,7], with only a handful of empirical studies published outside of China [8,9,10,11]. The focus of this study is to understand how internet use, online social capital, and locus of control relate to mental health. In a fundamentally uncontrollable pandemic, focusing on modifiable factors is essential to examine whether the negative mental health impact of COVID-19 could be buffered by other factors. If that is the case, then online social capital and internal locus of control could become the focus of future efforts to improve mental health, both in general, as well as in times of crisis.

Based on the scant knowledge available, we expect greater symptoms of anxiety, depression, and stress among those infected with COVID-19, among those who have family or friends who have become infected, and those who perceive themselves to be at high risk if they became infected. We also expect that online social capital will be protective against psychological distress [33,34,35,36]. However, it is currently unclear whether other internet variables, such as internet efficacy and internet experience, will relate to more or less distress. We also expect that individuals with a more external locus of control will report more distress. Likely those with a more internal locus of control will be more active when using the Internet, but the mental health implications of these are unclear. Finally, we expect greater psychological symptoms among young adults [2,4,8,13,32] and women [8,11,13,43].

## 2. Materials and Methods

### 2.1. Participants and Procedures

Participants were recruited from Amazon Mechanical Turk (MTurk). Inclusion criteria were that the participants needed to be 18 years old and residing in the United States, France, Germany, Italy, Spain or the United Kingdom. We chose these countries because, at the time of data collection, they were experiencing COVID-19 in significant numbers and had high responsive numbers of MTurk workers [44]. Two attention-checking items were used in the survey; participants who failed to respond correctly to them were removed. The final sample included 1723 participants, mean age 34.70 years (*SD* = 11.58 years), of whom 46.3% were female. Notably, 65.4% of the sample reported having higher education. When asked about employment status, 54.2% were working full time, 15.3% were working part time, 12.5% were unemployed, 11.2% were students and 6.8% had some other status. The data were collected from 23rd April to 7th May 2020. Prior studies have, indicated that MTurk data collected within the health sciences are both reliable and valid [45] and an efficient way to collect data [46]. The survey followed the APA ethical principles and code of conduct, and was carried out in accordance with the principles of the Declaration of Helsinki.

### 2.2. Measures

#### 2.2.1. Demographic Information

The survey included demographic questions on age, gender, subjective household income, current economic status and highest educational level attained.

In addition, the following measures were used:

#### 2.2.2. Depression Anxiety Stress Scale (DASS)

Symptoms of depression, anxiety and stress were assessed using the 21-item adaption of the 42-item version of the DASS instrument [47]. Each of the DASS subscales contains seven items, with response options ranging from 0 “did not apply to me at all” to 3 “applied to me very much or most of the time.” The subscales scores were calculated by summing the scores for the relevant seven items and multiplying by 2 to allow for comparison with DASS-42 [48]. The Cronbach’s *α* values for depression, anxiety and stress were 0.92, 0.89, and 0.91, respectively.

#### 2.2.3. COVID-19 Status

COVID-19 status was assessed using three dichotomous (Yes/No) questions; “Have you yourself been infected with COVID-19?”, “Has someone close to you (family member or close friend) been infected with COVID-19?” and “Would you consider yourself at high risk of serious illness or death if you were to become infected with COVID-19? For example, the elderly and those with underlying health issues could be in this group”.

#### 2.2.4. General Information Seeking and Information Seeking About COVID-19

Participants’ past experience searching for general health information on the Internet and specifically about COVID-19 was measured with two 4-item scales (Cronbach’s *α* = 0.92 and 0.92, respectively) [22]. Participants were asked how much they disagreed (1) or agreed (7) with the following statements: I have sought out health information on the Internet (about COVID-19), I have looked to different online sources to obtain health information (about COVID-19), I have paid close attention to health information on the Internet (about COVID-19), I have actively searched online for health information (about COVID-19). Total scores were averaged for both scales, with higher scores indicating more information seeking behavior, in general and about COVID-19.

#### 2.2.5. Internet Efficacy

Participants responded to four statements on their level of internet efficacy (Cronbach’s *α* = 0.89) [22]. The statements were adapted by Rains [49], from the Internet Self-Efficacy Scale [50], to obtain health information. Participants indicated on a 7-point scale ranging from 1 “strongly disagree” to 7 “strongly agree” how confident they were in their ability to: understand different procedures of accessing information on the Internet, understand how to use different search engines to gather health information on the Internet, evaluate the quality of different health websites, and find high quality health information on the Internet. Total scores were averaged for the scale, with higher scores indicating higher perceived self-efficacy in using the Internet to obtain health information.

#### 2.2.6. Internet Experience

Internet experience was assessed with four statements (Cronbach’s *α* = 0.90) [22,51]. Participants rated the following items on a 7-point scale from 1 “strongly disagree” to 7 “strongly agree”: I use the Internet often, I have a great deal of experience using the Internet, It is easy for me to access the Internet, I am familiar with the variety of information available on the Internet. Total scores were averaged for the scale, with higher scores denoting more perceived experience using the Internet.

#### 2.2.7. Internet Social Capital

Internet Social Capital was assessed using the Internet Social Capital Scale (ISCS) (Cronbach’s *α* = 0.91) [52]. Participants rated 20 statements on a 5-item scale from 1 “strongly agree” to 5 “strongly agree”, where we took care using the “online” distinction “There are several people online I trust to help solve my problems” for the scale. Total scores were averaged for the scale, with higher scores indicating higher internet social capital.

#### 2.2.8. Levenson Locus of Control Scale

Locus of control was assessed with the Levenson locus of control scale [53]. It is a 24-item instrument with three equal length subscales, powerful others, chance, and internal locus of control (with Cronbach’s *α* values 0.82, 0.82 and 0.73, respectively). Participants rated all statements on a 6-item scale, from −3 “strongly disagree” to +3 “strongly agree.” For each subscale, total scores were summed and added to 24. The powerful others and chance subscales encompass the degree to which participants believe that their fate is influenced by others or chance, with higher scores indicating a stronger external locus of control. Higher scores on the internal locus of control subscale indicate that personal factors are responsible for participants’ fate.

### 2.3. Data Analysis

To examine differences by COVID-19 groups (those infected, those with friends or family infected and those at risk), independent samples *t*-tests were used to examine differences in psychological symptoms. For the potentially interactive effects of COVID-19 infection and risk, a two-way ANOVA was used. To examine the relationships between the study variables, Pearson regression and multiple linear regression were used. SPSS version 26 was used to perform the analyses.

## 3. Results

For this sample, 57 (3.3%) individuals had become infected with COVID-19, 262 (15.3%) had a family member or close friend who had become infected, and 332 (19.3%) perceived that they were at high risk of serious illness if they were to become infected with the disease. The distribution of depression symptoms was such that 909 (52.8%) were categorized as normal, 171 (9.9%) as having mild symptoms, 282 (16.4%) as having moderate symptoms, 154 (8.9%) with severe symptoms and 207 (12.0%) with extremely severe symptoms. The distribution of anxiety symptoms was that 1056 (61.3%) were classified as normal, 93 (5.4%) as mild symptoms, 222 (12.9%) as having moderate symptoms, 95 (5.5%) with severe symptoms and 257 (14.9%) with extremely severe symptoms. For stress symptoms, 1121 (65.1%) were categorized as normal, 153 (8.9%) as having mild symptoms, 211 (12.2%) with moderate symptoms, 163 (9.5%) with severe symptoms and 75 (4.4%) with extremely severe symptoms.

As Table 1 shows, depression, anxiety, and stress were significantly greater for those who had become infected (depression: *t*(1715) = 4.18, *p* < 0.001, *d* = 0.54; anxiety: *t*(1715) = 7.94, *p* < 0.001, *d* = 0.91; stress: *t*(1715) = 4.23, *p* < 0.001, *d* = 0.55), for those with infected family members or friends (depression: *t*(1716) = 2.89, *p* = 0.004, *d* = 0.19; anxiety: *t*(1716) = 4.92, *p* < 0.001, *d* = 0.31; stress: *t*(1716) = 4.06, *p* < 0.001, *d* = 0.27), or for those who perceived themselves to be in a risk group (depression: *t*(1719) = 6.01, *p* < 0.001, *d* = 0.35); anxiety: *t*(1719) = 7.81, *p* < 0.001, *d* = 0.44; stress: *t*(1719) = 6.59, *p* < 0.001, *d* = 0.39), compared to those who had not, as revealed by independent samples *t*-tests.

A two-way ANOVA showed that, for depression and stress, significant elevating main effects on symptoms were found for both becoming infected with COVID-19 (depression: *F*(1, 1713) = 15.83, *p* < 0.001; stress: *F*(1, 1713) = 15.36, *p* < 0.001) and being in a risk group (depression: *F*(1, 1713) = 16.27, *p* < 0.001; stress: *F*(1, 1713) = 15.95, *p* < 0.001), but there was no interaction between illness and risk (depression: *F*(1, 1713) = 2.78, *p* = 0.10; stress: *F*(1, 1713) = 1.92, *p* = 0.17). However, for anxiety symptoms, being in a risk group was associated with significantly greater symptoms among those infected with COVID-19 (*F*(1, 1713) = 6.84, *p* = 0.01).

To examine the relationship between psychological symptoms, internet variables, locus of control, and age, we conducted a Pearson bivariate correlation (see Table 2). Internet efficacy and internet experience have negative relationships with psychological symptoms, and information seeking has a positive relationship with stress, but not the other symptoms. Internet social capital has a positive relationship with anxiety symptoms at the bivariate level, but not the other outcomes. An external locus of control (powerful others and chance) has a positive relationship with symptoms and an internal locus of control, a negative but weaker relationship with depression, anxiety, and stress symptoms. The relationship between internet efficacy, internet experience and information seeking was relatively strong and positive. Age had a significant but weak relationship with the other variables.

Multiple linear regression shows that, when controlling for demographic factors, becoming infected with COVID-19 and being at risk still relates to greater symptoms of depression, but having someone close infected does not (see Table 3). Internet experience and internet social capital relate to decreased depressive symptoms, but information seeking to greater symptoms. Having an internal locus of control is related to lower depression symptoms, but external loci to greater symptoms. Age continues to have a significant negative relationship with depression symptoms, so factors such as internet use and locus of control do not explain that difference. Similarly, being at risk for COVID-19 relates to greater symptoms, even when controlling for the other variables.

Table 3 also shows that COVID-19 infection and being at risk are related to more anxiety symptoms when controlling for demographic factors. Experience using the Internet has a negative relationship with anxiety symptoms, but internet efficacy has no relationship. Information seeking is related to more anxiety symptoms, especially general information seeking. The relationship between online social capital and anxiety is initially positive, but then disappears when controlling for locus of control. It therefore appears that locus of control may play a role in explaining both the relationship between social capital and anxiety symptoms, and between COVID-19 information seeking and anxiety symptoms. Internal locus of control has no relationship with anxiety symptoms, but external loci have positive relationships with symptoms. As with depression symptoms, age remains a significant covariate, showing that these variables do not explain age differences in psychological symptoms. The same analysis for stress symptoms can also be seen in Table 3. Women and younger people report greater stress, which is not explained by COVID-19, internet variables, or locus of control. Being at risk for COVID-19 relates to greater stress, as well as being infected with it. Experience with the Internet relates to less stress, but seeking information with more stress. Online social capital and internet efficacy do not relate to stress.

**Figure 1:**
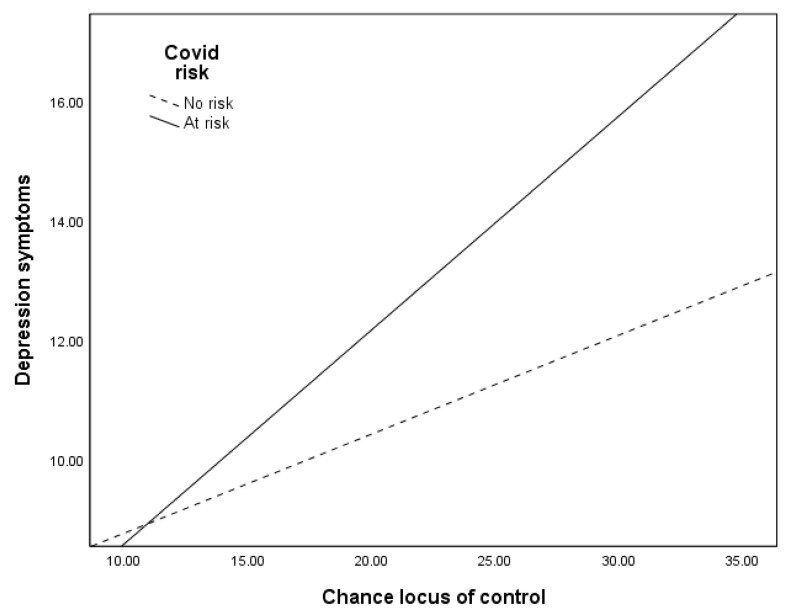
The relationship between chance locus of control and depression symptoms.

Multiple linear regressions showed that the relationships between COVID-19 risk and psychological symptoms depend on locus of control. The positive relationship between being at risk for COVID-19 and psychological symptoms only exists when external locus of control is high (see Table 4). When external locus of control is low, there is little or no relationship between COVID-19 risk and depression symptoms (significant interaction for powerful others locus of control (Δ*R*^2^ = 0.002, Δ*F*(1, 1689) = 5.56, *p* = 0.02, *b* = 0.14, *t*(1689) = 2.36, *p* = 0.02, and for chance locus of control (Δ*R*^2^ = 0.004, Δ*F*(1, 1689) = 9.87, *p* = 0.001, *b* = 0.19, *t*(1689) = 3.14, *p* = 0.001), for anxiety symptoms (Δ*R*^2^ = 0.002, Δ*F*(1, 1689) = 5.63, *p* = 0.02, *b* = 0.11, *t*(1689) = 2.37, *p* = 0.02, and for chance locus of control (Δ*R*^2^ = 0.003, Δ*F*(1, 1689) = 7.84, *p* = 0.005, *b* = 0.14, *t*(1689) = 2.80, *p* = 0.005) and for stress symptoms for chance locus of control (Δ*R^2^* = 0.004, Δ*F*(1, 1688) = 8.58, *p* = 0.003, *b* = 0.17, *t*(1688) = 2.93, *p* = 0.003), but was marginal for powerful others locus of control (Δ*R^2^* = 0.002, Δ*F*(1, 1688) = 3.32, *p* = 0.07, *b* = 0.10, *t*(1688) = 1.82, *p* = 0.07). When external locus rises, being at risk relates to greater symptoms. Figure 1 shows an example of these relationships, where the relationship between COVID-19 risk and depression symptoms becomes stronger as chance locus of control increases. Internal locus of control does not moderate the relationship between risk and psychological symptoms.

## 4. Discussion

Across these mental health outcomes, more exposure to COVID-19 translates to greater psychological symptoms, which is consistent with previous findings [12]. More specifically, being infected with COVID-19 is connected to greater symptoms of depression, anxiety and stress, as other studies have established [13,14]. Like previous work [19], we initially found elevated anxiety symptoms among those with infected family or friends, but these disappeared when controlling for demographic information. To our knowledge, this is the first study showing elevated psychological symptoms among those who perceive themselves to be in a risk group for COVID-19. Being at risk was consistently related to greater symptoms of depression, anxiety and stress. The uncertainty associated with risk group status, and especially in the context of becoming infected, is not surprising, as other studies have shown worries to relate to more anxiety and depression [5]. Moreover, the data show that being in a risk group and becoming infected with COVID-19 relate to significantly elevated symptoms of anxiety, which is previously unexplored in the COVID-19 literature.

The effects of the Internet and new media had differential associations with psychological symptoms, and these relationships are likely to be even more complex than this study was able to elucidate. For example, experience using the Internet is related to fewer psychological symptoms, meaning that promoting greater internet use in general could have beneficial health effects in a crisis. However, the correlational nature of the study makes it difficult to establish the ordering of these effects, and their relationship could perhaps be explained by some other risk factor, such as lower social status. On the other hand, seeking information on the Internet had significant positive relationships with symptoms, as similar work has shown [2,4,8,13,32]. It is unclear whether seeking information leads to subsequent mental health symptoms, or whether those who are experiencing negative feelings may seek out information to soothe themselves, creating a sort of feedback loop, where individuals that are worried look for information, and due to the lack of knowledge or changing knowledge, they feel worse and then look again. The currently limited, and continually growing, information available on the nature of the disease in terms of symptoms, methods of contagion, preventive efforts, treatment and life expectancy perhaps also contributes to a greater sense of uncertainty and need to look for the latest information available. Future studies must examine these relationships longitudinally to make it clear what types of interventions could be most effective.

Internet social capital seems to play a differential role depending on the psychological outcome. For depression, it relates to fewer symptoms, as previous literature would suggest [35,36]. However, internet social capital was not associated with stress symptoms. For anxiety, there was initially a positive relationship with internet social capital, which disappeared when introducing locus of control. Appel et al. [54] have questioned how well ISCS predicts social support, which is a predictive factor for both anxiety [55] and depression [56]. These findings therefore point to the need to further examine this concept in relation to mental health in the future, including its measurement.

The findings of the current study indicated that, during a global pandemic, locus of control remains a vital psychological attribute, where there was a more external locus related to greater symptoms and an internal locus to decreased symptoms, consistent with other work on the topic [26,27]. Furthermore, COVID-19 risk was only related to more mental health symptoms when external locus was medium or high, but not low. In an uncertain pandemic, feeling in control of one’s fate therefore appears to be protective against negative mental health impact. Promoting a more internal locus of control may be beneficial for mental health more generally, but especially during a worldwide crisis, such as the current pandemic.

### Limitations

The current findings are based on self-report from a self-selected MTurk sample and cannot be generalized to the population level. Some groups may have been severely impacted by the pandemic, but are unlikely to be represented in this sample, such as those with limited internet access. These results should be interpreted in light of that, showing that instead of fully representing the people in participating countries, these are first indications of these relationships during COVID-19. Relying on self-reporting can also be limiting, demanding that participants are fully able to estimate variables such as internet use and information seeking. Finally, the study design is correlational, making causal inference impossible. This becomes especially important in light of the findings on information seeking, where it is unclear whether seeking out information results in poorer mental health, or whether those who feel worse use news and information as a coping mechanism.

The findings of the study clearly show that exposure to COVID-19 relates to greater psychological symptoms, but this effect depends on exposure to the pandemic. The Internet has complex effects, where internet experience can be beneficial, but that continually searching for health information may be harmful for mental health. Authorities and public health officials therefore need to promote using the Internet, but at the same time warn against its negative effects if constantly consumed. The effects of media remain when controlling for locus of control, suggesting that the effects of media use cannot be directly explained by promoting greater feelings of control. Moreover, psychological symptoms were higher among younger people, which was not explained with COVID-19, internet use or locus of control, making it essential to understand better why younger adults are at particular risk for distress. Moving forward, public health authorities need to put a specific focus on investing in mental health care workers, to provide support post-disease, as well as to prevent serious long-term mental health consequences of the outbreak, as previous epidemics have shown [57].

## 5. Conclusions

Being infected with COVID-19 or being at high risk if infected were related to greater depression, anxiety and stress. Experience using the Internet was related to fewer symptoms, but seeking information was related to greater symptoms. Online social capital was related to fewer depressive symptoms. The relationship between COVID-19 exposure and mental health is only present among those who are high or medium in external locus of control.

## Figures and Tables

**Table 1:** Psychological symptoms by COVID-19 status.

Mental Health	Infected		Family/Friend Infected		In Risk Group	
	Yes	No	Yes	No	Yes	No
	M(SD)	M(SD)	M(SD)	M(SD)	M(SD)	M(SD)
Depressive Symptoms	17.23 (11.85) **	11.04 (10.96)	13.08 (11.46) *	10.94 (10.95)	14.57 (12.27) **	10.50 (10.60)
Anxiety Symptoms	17.37 (12.31) **	7.48 (9.12)	10.44 (10.63) **	7.34 (9.11)	11.40 (11.07) **	6.98 (8.78)
Stress Symptoms	17.89 (11.08) **	11.97 (10.37)	14.56 (10.69) **	11.73 (10.34)	15.54 (11.17) **	11.39 (10.11)

* *p* < 0.05, ** *p* < 0.001.

**Table 2:** Correlation between psychological symptoms, internet variables, locus of control and age.

Variable name	1	2	3	4	5	6	7	8	9	10	11	12	M(SD)
1. Depression symptoms	1	0.73 **	0.81 **	−0.08 *	−0.17 **	0.03	0.05	−0.02	−0.24 **	0.43 **	0.42 **	−0.17 **	11.28 (11.05)
2. Anxiety symptoms	-	1	0.80 **	−0.11 **	−0.29 **	0.01	0.05	0.10 **	−0.12 **	0.46 **	0.48 **	−0.18 **	7.83 (9.42)
3. Stress symptoms	-	-	1	−0.05 *	−0.14 **	0.08 *	0.12 **	0.04	−0.15 **	0.42 **	0.41 **	−0.17 **	12.19 (10.45)
4. Internet efficacy	-	-	-	1	0.58 **	0.52 **	0.34 **	0.11 **	0.26 **	−0.03	−0.09 **	0.10 **	5.81 (1.03)
5. Internet experience	-	-	-	-	1	0.38 **	0.31 **	0.04	0.21 **	−0.13 **	−0.16 **	0.06 *	6.52 (0.79)
6. Information seeking general	-	-	-	-	-	1	0.59 **	0.19 **	0.17 **	0.09 **	0.02	0.16 **	5.54 (1.28)
7. Information seeking COVID-19	-	-	-	-	-	-	1	0.14 **	0.09 **	0.09 **	0.05 *	0.05 *	5.61 (1.38)
8. Internet social capital	-	-	-	-	-	-	-	1	0.21 **	0.12 **	0.14 **	−0.09 **	3.30 (0.66)
9. Internal locus of control	-	-	-	-	-	-	-	-	1	−0.15 **	−0.19 **	0.16 **	32.73 (7.06)
10. Powerful others locus of control	-	-	-	-	-	-	-	-	-	1	0.74 **	−0.09 **	22.92 (9.31)
11. Chance locus of control	-	-	-	-	-	-	-	-	-	-	1	−0.15 **	21.87 (9.24)
12. Age	-	-	-	-	-	-	-	-	-	-	-	1	34.70 (11.58)

* *p* < 0.05, ** *p* < 0.001.

**Table 3:** Multiple linear regression predicting depression, anxiety and stress symptoms, showing standardized coefficients.

Predictors	Depression				Anxiety				Stress			
	Model 1	Model 2	Model 3	Model 4	Model 1	Model 2	Model 3	Model 4	Model 1	Model 2	Model 3	Model 4
Gender	0.03	0.02	0.02	0.02	0.02	0.01	0.02	0.02	0.09 **	0.08 *	0.07 *	0.08 **
Age	−0.16 **	−0.19 **	− 0.20 **	−0.12 **	−0.17 **	−0.21 **	−0.20 **	−0.13 **	−0.17 **	−0.20 **	−0.20 **	−0.14 **
Income	0.09 **	0.08 **	0.08 *	0.03	−0.01	−0.03	−0.02	−0.05 *	0.03	0.02	0.02	−0.02
Economic status	0.03	0.02	0.04	0.05 *	−0.04	−0.04	−0.01	0.01	0.00	−0.01	0.02	0.03
Education	−0.04	−0.03	−0.03	−0.03	−0.07 *	−0.05 *	−0.05 *	−0.05 *	−0.04	−0.03	−0.03	0.03
Contracted COVID-19	-	0.07 *	0.06 *	0.03	-	0.13 **	0.10 **	0.08 **	-	0.07 *	0.05 *	0.03
Someone close COVID-19	-	0.02	0.02	0.03	-	0.05 *	0.05 *	0.05 *	-	0.05	0.04	0.05 *
At risk for COVID-19	-	0.16 **	0.14 **	0.08 **	-	0.21 **	0.18 **	0.13 **	-	0.17 **	0.16 **	0.10 **
Internet efficacy	-	-	−0.01	0.03	-	-	0.00	0.02	-	-	−0.03	−0.10
Internet experience	-	-	−0.21 **	−0.12 **	-	-	−0.35 **	−0.28 **	-	-	−0.20 **	−0.12 **
Information seeking general	-	-	0.09 *	0.06 *	-	-	0.09 *	0.05	-	-	0.10 *	0.07 *
Information seeking COVID-19	-	-	0.07 *	0.04	-	-	0.09 *	0.06 *	-	-	0.12 **	0.09 **
Internet social capital	-	-	−0.05 **	−0.08 **	-	-	0.39 *	0.00	-	-	−0.03	−0.04
ILC	-	-	-	−0.12 **	-	-	-	0.00	-	-	-	−0.05 *
POLC	-	-	-	0.23 **	-	-	-	0.19 **	-	-	-	0.23 **
CLC	-	-	-	0.19 **	-	-	-	0.24 **	-	-	-	0.17 **
R2	3.8% **	6.8% **	10.6% **	26.9% **	3.2% **	10.0% **	20.2% **	34.7% **	3.6% **	7.5% **	11.9% **	25.2% **

* *p* < 0.05, ** *p* < 0.001. ILC = internal locus of control, POLC = powerful others locus of control, CLC = chance locus of control.

**Table 4:** The relationship between risk of COVID-19 at different levels of external locus of control.

Level of Locus of Control	Depression Symptoms	Anxiety Symptoms	Stress Symptoms
Chance locus of control	-	-	-
Low	0.30	1.52 *	0.74
Mean	2.19 *	2.89 **	2.45 *
High	3.88 **	4.12 **	3.99 **
Powerful others locus of control	-	-	-
Low	0.89	1.81 *	1.54
Mean	2.27 *	2.92 **	2.56 **
High	3.50 **	3.93 **	3.48 **

* *p* < 0.05, ** *p* < 0.001.
